# Childhood leukaemia: long-term excess mortality and the proportion ‘cured’

**DOI:** 10.1038/sj.bjc.6604466

**Published:** 2008-07-01

**Authors:** A Shah, C A Stiller, M G Kenward, T Vincent, T O B Eden, M P Coleman

**Affiliations:** 1 Non-communicable Disease Epidemiology Unit, Department of Epidemiology and Population Health, London School of Hygiene and Tropical Medicine, Keppel Street, London WC1E 7HT, UK; 2 Childhood Cancer Research Group, Department of Paediatrics, University of Oxford, 57 Woodstock Rd, Oxford OX2 6HJ, UK; 3 Academic Unit of Paediatric and Adolescent Oncology, Teenage Cancer Trust Young Oncology Unit, Christie Hospital NHS Trust, Wilmslow Road, Manchester M20 4BX, UK

**Keywords:** survival, cure, childhood leukaemia, trends

## Abstract

Survival from childhood leukaemia has increased, but the proportion of children cured is unknown. The proportion ‘cured’ is defined as the proportion of survivors for whom, as a group, there is no longer excess mortality compared to the general population. Average time to cure is defined as the time since diagnosis at which the excess mortality rate has declined to or below a predetermined small value. Data on children diagnosed with leukaemia during 1971–2000 in Great Britain were used to estimate trends in survival, the proportion cured and the average time to cure. Five-year survival for all types of leukaemia combined rose from 33 to 79% by 2000. The percentage cured rose from 25 to 68% by 1995; it is predicted to increase to 73% for those diagnosed more recently. Average time to cure increased from 12 years (95% confidence interval (CI): 11–14) to 19 years (95% CI: 14–26) for lymphoid leukaemia (average annual increase of 0.3 years; *P*<0.001), but remained at about 5 years for acute nonlymphoblastic leukaemia. The proportion of children cured of leukaemia has risen dramatically, but the period of excess mortality associated with lymphoid leukaemia has also increased, possibly because of late relapse, secondary malignancy and toxicity from treatment.

Leukaemia accounts for approximately one-third of all childhood malignancies in Great Britain (Swerdlow *et al*, 2001). Over 400 cases of childhood leukaemia are diagnosed every year, of which lymphoid leukaemia represents 80% and the remainder are mostly acute nonlymphoblastic leukaemia (ANLL). Survival from childhood leukaemia at 5 years after diagnosis has risen dramatically, from less than 5% in the early 1960s (Thames Cancer Registry, 1994) to 79% for children diagnosed during 1996–2000 (Stiller *et al*, 2007).

The increase in survival is attributed to the use of intensive chemotherapy, combined with other modalities of treatment, and to improved supportive care (Eden *et al*, 2000; Gibson *et al*, 2005). The number of childhood leukaemia survivors who are alive more than 10 years after diagnosis has increased as a result, and there are currently over 5500 long-term survivors in Great Britain. However, intensive therapies for childhood leukaemia have long-term adverse effects, including continuing excess mortality beyond 10 years after diagnosis (Hawkins, 1989). Therefore, the proportion of children who are actually ‘cured’, that is, who have a normal life expectancy after a diagnosis of leukaemia, is not known.

## Materials and methods

Data for children who were diagnosed with leukaemia in Great Britain during 1971–2000 and followed up until the end of 2004 were obtained from the National Registry of Childhood Tumours (NRCT), which is managed by the Childhood Cancer Research Group (CCRG).

The NRCT includes virtually all children resident in Great Britain who are diagnosed with a malignant neoplasm under the age of 15 years. The methods of ascertainment to obtain registrations of children from a variety of population-based sources by CCRG are described elsewhere (Stiller *et al*, 2007). The Childhood Cancer Research Group has received information from its various sources on an estimated 99% of all cases of childhood leukaemia diagnosed since 1969.

Childhood cancer patients are routinely flagged at the National Health Service Central Registers (NHSCR) for England and Wales and for Scotland approximately 5 years after diagnosis. The Childhood Cancer Research Group is then notified of any subsequent cancer registrations, or of the death or emigration of any of these patients. Information on the vital status of all children treated by the Children's Cancer and Leukaemia Group who are not yet flagged at NHSCR is also received annually by CCRG. Flagged patients for whom no death or emigration details had been recorded were assumed to be alive on 31 December 2004. Leukaemia was classified according to the International Classification of Childhood Cancer (Kramárová *et al*, 1996).

A suite of programmes was used to assess data quality, by filtering each record through a standard series of checks (Coleman *et al*, 1999). Records were considered to be ineligible if they were incomplete, if they had ineligible morphology codes or were duplicates. Records were checked for impossible sequences of dates of birth, diagnosis and follow-up or death, and for zero survival. Patients with zero survival were defined as those who were diagnosed and died on the same day, whose leukaemia was only detected at postmortem, or who were notified only from a death certificate (DCO), the last group because date of diagnosis is unknown.

The population-based data obtained from the NRCT were of very high quality. Less than 3% of children registered with leukaemia were lost to follow-up by the end of 2004. No duplicates or invalid sequences of dates were identified, and only 0.8% of patients were excluded because the gender was unknown or they were diagnosed at postmortem or registered only through a death certificate or because no follow-up was available after diagnosis.

Relative survival and the proportion cured were estimated for children diagnosed with all types of leukaemia combined and separately for lymphoid leukaemia and ANLL. Relative survival is the ratio of the survival observed in a group of cancer patients and the survival that would have been expected if they had only been subjected to the background mortality observed in the general population. It was estimated using a maximum likelihood approach for individual records (Estève *et al*, 1994) using the STATA algorithm ***strel*** (Coleman *et al*, 1999). Expected survival was estimated by applying the background mortality rates in the general population by age, sex and time period to the patient population. Background mortality rates by sex and single year of age at death up to age 99 were derived from life tables constructed by the Government Actuary's Department for Great Britain from 1980 onwards.

**Figure 1:**
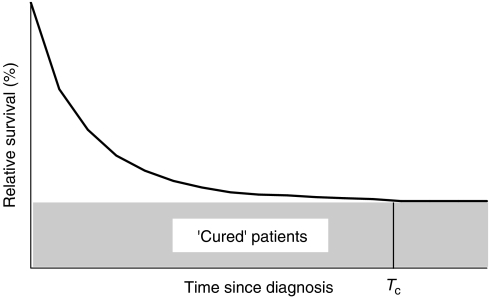
The schematic representation of the proportion of patients who are ‘cured’ (shaded area) and the average time since diagnosis at which cure can be declared (Tc).

The relative survival function tends to reach a plateau at some point after diagnosis; this plateau represents the proportion of survivors for whom, as a group, there is no longer any excess mortality compared with the general population. The value of the function at the time point when this plateau is reached can be taken as an estimate of the proportion of patients who are cured of their disease (Figure 1). This approach can be used to derive a further indicator, the average duration of time since diagnosis at which cure can reasonably be declared (see Appendix for equations). The percentage of children cured of leukaemia, by period of diagnosis, was modelled from relative survival estimates up to 20 years after diagnosis. As this approach refers to a population, some long-term survivors will still die from their cancer, and some patients who die before the time point at which cure is declared (‘fatal’ cases) would have died from other causes.

The risk of death for ‘fatal’ cases was modelled as Weibull-distributed with time since diagnosis (Verdecchia *et al*, 1998). Average time to cure is estimated as the time at which an arbitrary but small proportion of ‘fatal’ cases is still alive. In this study, following a sensitivity analysis, average time to cure was defined as the point at which 1% of ‘fatal’ cases were still alive (De Angelis *et al*, 1999). The average time since diagnosis at which cure can reasonably be declared is sensitive both to the sample size and to the (arbitrary) percentage of ‘fatal’ cases still alive that is used to determine the average time of cure. Formulae for the variance of the average time to cure were developed to be able to assess the variability of average time to cure in each analysis (see Appendix). A nonlinear least squares method was used to fit the Weibull model to the relative survival values by time since diagnosis. The standard error was estimated using a standard linear approximation (Kendall and Stuart, 1969). Variance-weighted least squares linear regression was used to evaluate time trends in survival, in the percentage of children cured and in the average time to cure.

The period approach (Brenner and Gefeller, 1997) was used to predict long-term relative survival (up to 20 years) for children diagnosed during the 2-year period 1999–2000. This ‘predicted’ survival was then used to estimate the percentage of children diagnosed during 1999–2000 who were expected to be cured, using the Weibull model applied to follow-up data accrued during the period 1999–2004.

## Results

Survival for children with leukaemia has improved significantly since 1971 (Table 1). For all types of leukaemia combined, 5-year survival increased from 33% for children diagnosed during 1971–1975 to 79% for those diagnosed during 1996–2000. This represents an estimated (linear) average increase of 1.9% every year over the 25-year period between the midpoints of these quinquennia.

Since 1971, survival for children with lymphoid leukaemia has consistently been much higher than that for children diagnosed with ANLL (Table 1). Five-year relative survival was 83% for children diagnosed during 1996–2000 with lymphoid leukaemia and 66% for those with ANLL. The average annual improvement in survival over the entire period was higher for ANLL (2.4%) than for lymphoid leukaemia (1.7%), although survival for ANLL started from a much lower value.

Taking all types of leukaemia combined as a group, the percentage of children who appear to have been cured has increased significantly, from 25% for children diagnosed during 1971–1975 to 68% for children diagnosed during 1991–1995 (Table 1), and it is predicted to increase to 73% for those most recently diagnosed. Average time to cure increased from 11.0 years (95% confidence interval (CI): 10–12) in children diagnosed during 1971–1975 to 15.9 years (95% CI: 12–21) in children diagnosed during 1986–1990 (average annual increase of 0.2 years; *P*<0.001). In contrast to the steady improvement in the percentage cured, the average time at which cure was declared increased most rapidly during the 1980s.

**Figure 2:**
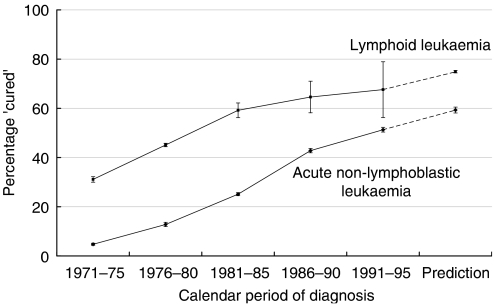
Trends in the percentage of children ‘cured’ of lymphoid leukaemia and acute nonlymphoblastic leukaemia: Great Britain, children diagnosed during 1971–1995. The predictions of the proportion cured were made for children diagnosed during 1999–2000 and are based on follow-up accrued during 1999–2004.

**Figure 3:**
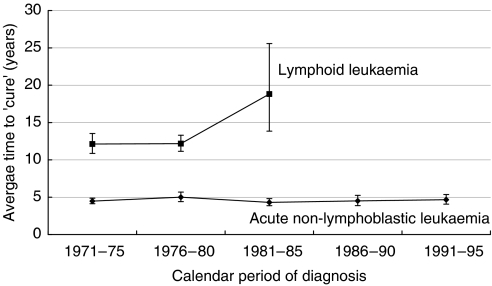
Trends in the average time to ‘cure’ for children with lymphoid leukaemia and acute nonlymphoblastic leukaemia by period of diagnosis: Great Britain, children diagnosed during 1971–1995.

The percentage of children cured is predicted to increase to 75% for lymphoid leukaemia and 59% for ANLL (Figure 2). The average duration of time since diagnosis at which cure can reasonably be declared has increased from 12 years (95% CI: 11–14) to 19 years (95% CI: 14–26) for lymphoid leukaemia (average annual increase of 0.3 years; *P*<0.001), but remained at about 5 years for ANLL (Figure 3).

## Discussion

Survival and the proportion cured of childhood leukaemia have both increased dramatically since the 1970s. But children with lymphoid leukaemia, as a group, now experience excess mortality for at least 19 years after diagnosis. Given this evidence of a prolonged period of excess mortality, estimates of cure remain relevant today for lymphoid leukaemia survivors who were diagnosed many years ago.

The robustness of estimates of cure for children diagnosed in more recent years is dependent on the length of time before the plateau of relative survival occurs and whether sufficient follow-up time has been observed for the plateau to be detected. Estimates of cure become less precise as the duration of available follow-up data becomes shorter, although each child was followed for at least 9 years. When excess mortality continues beyond the most recent time point for which follow-up data are available, the percentage cured is derived by extrapolation of the cure model. This was done for children diagnosed with lymphoid leukaemia during 1991–1995.

The prediction of cure for lymphoid leukaemia has much narrower CIs than the estimate of cure for children diagnosed during 1991–1995, because follow-up data for children diagnosed during the 22 years 1979–2000 contributed to the period analysis, whereas only 13 years of follow-up were available for children diagnosed during 1991–1995. Period analysis provides the most robust approach to predicting outcome for children diagnosed most recently, but it is still only of limited value where new treatment regimens have been introduced. The impact of these new treatment regimens cannot yet be incorporated into long-term survival predictions, because follow-up data are insufficient.

Remarkable improvements have been made in treating and ‘curing’ leukaemia since 1971. The evidence for risk-directed therapy underpinning these huge gains in survival from childhood leukaemia derives from an almost continuous series of national trials that began in 1970, funded by the Medical Research Council (MRC) (MRC Working Party on Leukaemia in Childhood, 1986; Eden *et al*, 2000; Gibson *et al*, 2005). All the studies that have examined long-term survival in childhood cancer patients, in Britain and in other countries, have concluded that survivors experienced excess mortality beyond 5 years after diagnosis, with a 10.8-fold excess in all-cause mortality in the United States and Nordic countries (Hawkins, 1989; Robertson *et al*, 1994; Mertens *et al*, 2001; Möller *et al*, 2001; Cardous-Ubbink *et al*, 2004).

These results may appear paradoxical, in that survival and the proportion of children cured have both increased and excess mortality has fallen, but excess mortality now lasts on average 7 years longer than it used to. Likely reasons for the increased duration of excess mortality include an increase in the occurrence of late relapse and of second cancers, as well as increased toxicity of treatment and prolonged survival of ‘fatal’ cases (Vora *et al*, 1998; Robison and Bhatia, 2003; Pui *et al*, 2005). In the Nordic countries, 300 deaths were seen in 5-year survivors of childhood leukaemia; 85% of these were due to the primary leukaemia, 11% due to the treatment for the first cancer and 3% died from a second primary cancer (Möller *et al*, 2001).

Modern therapy has increased event-free survival, but late relapse still occurs (Vora *et al*, 1998; Lawson *et al*, 2000; Roy *et al*, 2005; Feltbower *et al*, 2007), although it can be unclear whether this is a ‘true’ relapse of the original leukaemia or the occurrence of a second similar malignancy. It has been postulated, for example, that a persistent preleukaemic clone may survive chemotherapy for the original disease and later lead to a second, similar leukaemia, after further events induce disease progression (Vora *et al*, 1998).

Radiotherapy and chemotherapy are associated with serious complications, including cardiotoxicity, endocrinological effects, neurocognitive and neuropsychological effects and second malignancies (Neglia *et al*, 1991; Hawkins *et al*, 1996; Pui *et al*, 2003; Robison and Bhatia, 2003; Jenkinson *et al*, 2004; Oeffinger *et al*, 2006). The risk of a second cancer has been found to be highest in children who received irradiation therapy or suffered a relapse, and this risk persists for up to 30 years after treatment (Kimball Dalton *et al*, 1998; Lonig *et al*, 2000; Bhatia *et al*, 2002).

It remains to be seen whether the abandonment of cranial irradiation therapy from the late 1990s (Hill *et al*, 2004) will lead to a reduction in the occurrence of central nervous system tumours in leukaemia survivors, and whether the reduction in total dose of therapeutic agents, such as anthracylines, will reduce the occurrence of cardiovascular complications.

The average length of time to cure is markedly shorter for ANLL than for lymphoid leukaemia. A likely reason is that most relapses occur early in ANLL and the overall survival is lower than that for lymphoid leukaemia: survival beyond 5 years is thus virtually equivalent to actual cure. As there are far fewer long-term survivors for ANLL than for lymphoid leukaemia, we are not yet in a position to assess long-term treatment-related toxicity in ANLL beyond 5 years after diagnosis.

Excess morbidity and mortality in survivors of childhood lymphoid leukaemia emphasise the need for long-term medical surveillance of these children. Trends in the percentage of children who can be considered cured of their leukaemia and the average time since diagnosis at which cure can reasonably be declared provide an additional approach to evaluating the long-term outcome of leukaemia. These measures usefully supplement estimates of 5-year survival, the traditional measure of prognosis.

## Figures and Tables

**Table 1:** Survival and ‘cure’ trends for childhood leukaemia: Great Britain, children diagnosed during 1971–2000

	**Calendar period of diagnosis**																				
	**1971–1975**			**1976–1980**			**1981–1985**			**1986–1990**			**1991–95**			**1996–2000**			**Average annual change^a^**		
All types		95% CI			95% CI			95% CI			95% CI			95% CI			95% CI			95% CI	
Number of patients	2379			2136			1942			2080			2215			2317					
Five-year relative survival (%)	32.8	30.9	34.7	46.0	43.8	48.1	61.2	59.0	63.3	68.6	66.5	70.5	76.1	74.3	77.8	79.0	77.2	80.7	1.9	1.8	1.9
Percentage ‘cured’	24.7	23.8	25.7	38.7	38.1	39.4	54.1	53.1	55.0	61.4	59.5	63.4	67.5	64.9	70.1	NA	NA	NA	2.5	2.5	2.5
Average time to ‘cure’ (years)	11.0	9.9	12.3	11.2	10.2	12.2	12.8	11.2	14.7	15.9	12.2	20.7	FU	FU	FU	NA	NA	NA	0.2	0.2	0.3
																					
*Lymphoid*																					
Number of patients	1827			1726			1573			1675			1822			1873					
Five-year relative survival (%)	40.6	38.3	42.8	53.3	50.9	55.6	69.4	67.1	71.6	74.7	72.5	76.7	81.3	79.4	83.0	82.6	80.8	84.3	1.7	1.6	1.7
Percentage ‘cured’	31.1	30.0	32.3	45.1	44.4	45.8	59.2	56.2	62.2	64.6	58.2	71.1	67.6	56.3	79.0	NA	NA	NA	2.6	2.6	2.6
Average time to ‘cure’ (years)	12.1	10.9	13.5	12.2	11.1	13.3	18.8	13.8	25.6	FU	FU	FU	FU	FU	FU	NA	NA	NA	0.3	0.3	0.3
																					
*ANLL*																					
Number of patients	425			338			298			321			329			349					
Five-year relative survival (%)	5.5	3.7	8.0	14.3	10.8	18.2	26.3	21.5	31.4	44.6	39.1	50.0	51.9	46.4	57.2	65.8	60.4	70.6	2.4	2.2	2.6
Percentage ‘cured’	4.7	4.3	5.2	12.8	11.9	13.6	25.1	24.5	25.7	42.8	41.9	43.6	51.3	50.4	52.3	NA	NA	NA	2.4	2.4	2.4
Average time to ‘cure’ (years)	4.5	4.1	4.9	5.0	4.4	5.7	4.3	3.9	4.8	4.5	3.9	5.3	4.7	4.1	5.3	NA	NA	NA	0.0	0.0	0.0

ANLL=acute nonlymphoblastic leukaemia; CI=confidence interval.

FU – follow-up data were inadequate for estimating time to ‘cure’; NA – not available: estimates of ‘cure’ were not produced for the 1996–2000 period of diagnosis because the available follow-up was too short.

a Average annual increase over 25 years (1971–2004) for survival and over 20 years (1971–1995) for ‘cure’. The estimate of trend was based on the midpoint of each of the intervals.

## Appendix

In the Weibull mixture survival model used to estimate ‘cure’, the cumulative relative survival (*S*) is given by:

where *P* represents the proportion of patients ‘cured’, *Q* represents the proportion of ‘fatal’ cases, *λ* is the excess hazard, *t* is the time since diagnosis and *β* is the ‘shape’ parameter, indicating change in the excess hazard with time since diagnosis (Verdecchia *et al*, 1998). The Weibull distribution includes *β*, so if *β* is less than 1, the excess hazard (*λ*) is decreasing with time, and if *β* is greater than 1, the excess hazard is increasing.

The proportion of ‘cured’ cases (*P*) is estimated from the intercept of this model. The function (*Q*)exp(−*λt*)^*b*^ models relative survival until a plateau occurs.

Average time to ‘cure’ (*T*_c_) provides the time at which only a proportion, *α*, of ‘fatal’ cases are still alive. In this work, α was taken to be 1%, following an evaluation of the estimate of average time to ‘cure’ to the proportion of ‘fatal’ cases remaining alive. De Angelis *et al* (1999) define average time to ‘cure’ as:

A standard Taylor series approximation was used to obtain the variance of average time to ‘cure’ (Kendall and Stuart, 1969). The formulae, which were programmed in STATA, are given below:

where Var is the variance of each parameter and cov is the covariance of the stated parameters. Then the variance of average time to ‘cure’ is given by:

and the 95% confidence interval around average time to ‘cure’ is given by a standard Normal approximation:
